# Extracorporeal Membrane Oxygenation for Respiratory Failure: A Narrative Review

**DOI:** 10.3390/jcm13133795

**Published:** 2024-06-28

**Authors:** John C. Grotberg, Daniel Reynolds, Bryan D. Kraft

**Affiliations:** Division of Pulmonary and Critical Care Medicine, Department of Medicine, Washington University School of Medicine, Saint Louis, MO 63131, USA; danielreynolds@wustl.edu (D.R.); kraft@wustl.edu (B.D.K.)

**Keywords:** extracorporeal membrane oxygenation, respiratory insufficiency, respiratory distress syndrome

## Abstract

Extracorporeal membrane oxygenation support for respiratory failure in the intensive care unit continues to have an expanded role in select patients. While acute respiratory distress syndrome remains the most common indication, extracorporeal membrane oxygenation may be used in other causes of refractory hypoxemia and/or hypercapnia. The most common configuration is veno-venous extracorporeal membrane oxygenation; however, in specific cases of refractory hypoxemia or right ventricular failure, some patients may benefit from veno-pulmonary extracorporeal membrane oxygenation or veno-venoarterial extracorporeal membrane oxygenation. Patient selection and extracorporeal circuit management are essential to successful outcomes. This narrative review explores the physiology of extracorporeal membrane oxygenation, indications and contraindications, ventilator management, extracorporeal circuit management, troubleshooting hypoxemia, complications, and extracorporeal membrane oxygenation weaning in patients with respiratory failure. As the footprint of extracorporeal membrane oxygenation continues to expand, it is essential that clinicians understand the underlying physiology and management of these complex patients.

## 1. Introduction

Mortality in the intensive care unit (ICU) remains high for patients with acute respiratory failure despite conventional life-support modalities, such as noninvasive ventilation (NIV) and invasive mechanical ventilation (IMV) [1,2]. For select patients with severe hypoxemia or hypercapnia refractory to conventional support [3], extracorporeal membrane oxygenation (ECMO) has emerged as an effective intervention [4,5]. Advances in ECMO technology have improved safety and efficacy, and allowed for broader utilization of ECMO for respiratory failure [6]. While the most common use of ECMO is for acute respiratory distress syndrome (ARDS), there is increasing use for other etiologies of respiratory failure as well (Table 1).

ECMO has been an available treatment for respiratory failure since the 1970s [7], though its application has dramatically increased over the last 20 years. Since the H1N1 pandemic of 2009, yearly ECMO runs for respiratory failure have increased by nearly 800%, from about 500 runs per year to more than 4000 runs per year globally [8]. More recently, cases surged again between 2020 and 2022 due to the COVID-19 pandemic, but have since returned to pre-COVID numbers. Despite advances in care, survival to hospital discharge in patients supported on ECMO for respiratory failure remains at 57% [8].

ECMO offers many (theoretical) physiologic benefits, including facilitating ultra-low tidal volume (V_T_) ventilation (i.e., V_T_ ≤ 4 mL/kg ideal body weight [IBW]) in ARDS or massive air leak syndromes (e.g., due to bronchopleural fistula) and providing extracorporeal CO_2_ removal in severe asthma exacerbations. Most patients with respiratory failure requiring ECMO can be supported with a veno-venous (V-V) ECMO configuration alone, and do not require cardiac mechanical support as well, although select patients with right ventricular (RV) failure may benefit from alternative configurations such as veno-pulmonary (V-P) ECMO, which will be discussed later. Given the increased popularity of ECMO support in the wake of the COVID-19 pandemic, this review will focus on the indications, physiology, and management of the patient requiring ECMO support for respiratory failure.

## 2. Nomenclature and Configurations

In an effort to standardize nomenclature for extracorporeal life support (ECLS), the extracorporeal life support organization (ELSO) recently published a position paper on the preferred abbreviations and nomenclature for ECMO cannula configuration. Briefly, ECMO modes, such as veno-venous and venoarterial are abbreviated without hyphens respectively as VV ECMO and VA ECMO. ECMO configurations are abbreviated with capital letters starting with drainage site on the left with movement of flow toward the membrane lung and subsequently to the return site. The membrane lung is represented by a hyphen. For example, V-V ECMO would represent venous drainage to the membrane lung followed by venous return, V-P ECMO would represent venous drainage to the membrane lung followed by venous return to the pulmonary artery, and V-A ECMO would represent venous drainage to the membrane lung followed by arterial return. An additional venous drainage cannula in V-V ECMO would be represented as VV-V ECMO, while an additional venous return cannula in V-A ECMO would be represented as V-AV ECMO. Further levels of hierarchy beyond configuration include lower case letters to represent minor flow cannulas, cannulation sites, and cannula tip positions [9].

**Figure 1:**
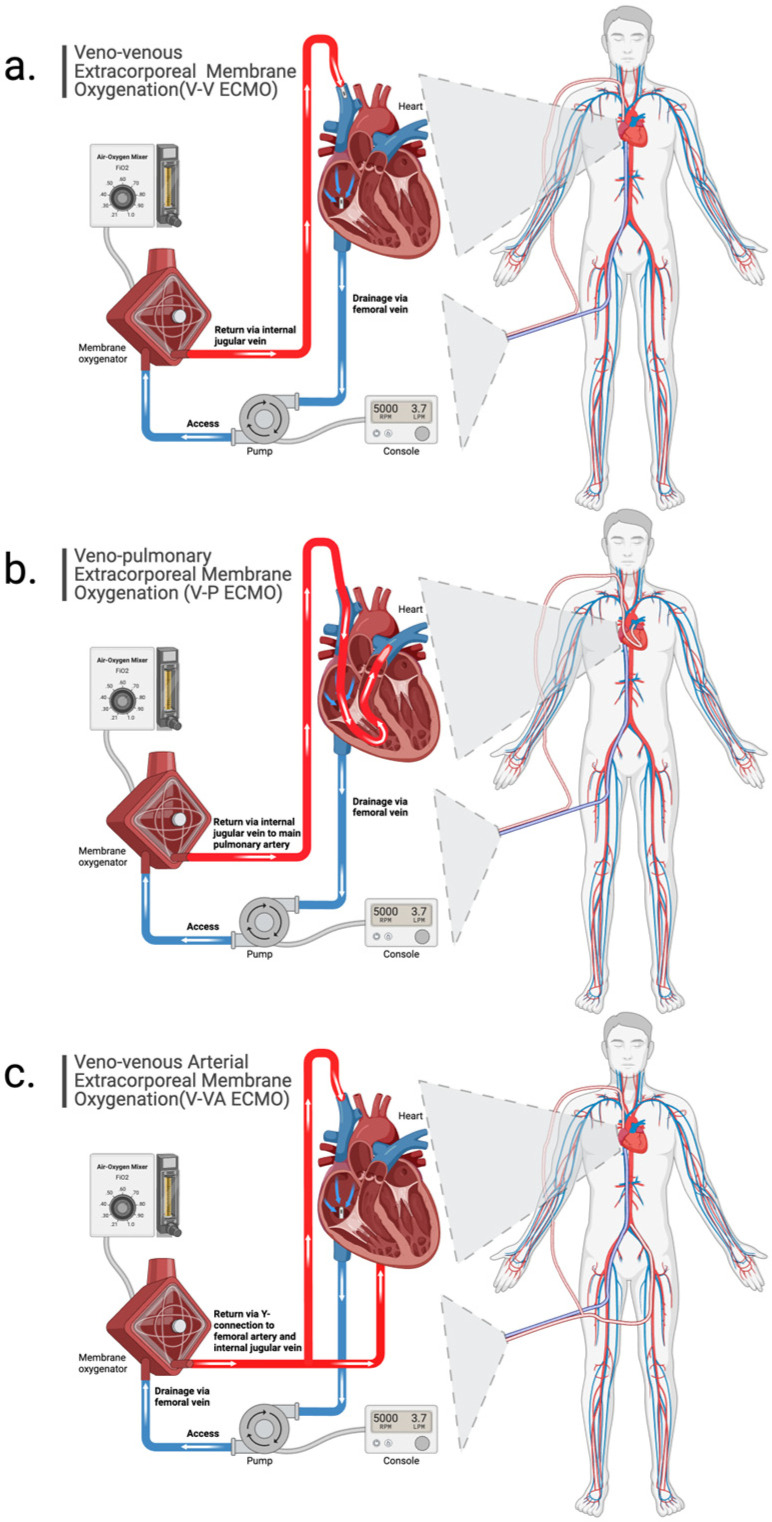
Schematic demonstrating configurations for (**a**) veno-venous (V-V) ECMO, (**b**) veno-pulmonary (V-P) ECMO, and (**c**) veno-venoarterial (V-VA) ECMO.

As previously mentioned, respiratory failure is most commonly supported with VV ECMO modes. Typically, the V-V ECMO configuration consists of a venous drainage cannula in the intrahepatic IVC and a venous return cannula in the superior vena cava (SVC) via the internal jugular vein, ideally about 10–15 cm apart to reduce the risk of recirculation. V-V ECMO may also be accomplished with a single, dual-lumen cannula placed via the internal jugular vein with the outflow tip seated in the right atrium directing return flow across the tricuspid valve. Some patients, however, may require cardiac support as well, and an additional return cannula may be placed in the femoral artery (V-VA ECMO). In this scenario, post-membrane lung flow is split between venous and arterial return cannulas (Figure 1). Acute cor pulmonale is highly prevalent in patients with acute respiratory distress syndrome (~22%), and patients with severe acute cor pulmonale have increased mortality risk [10,11]. In the wake of the COVID-19 pandemic, there has been more interest in the V-P ECMO configuration for right ventricular support. V-P ECMO may be accomplished as a two-cannula strategy, with drainage cannula in the intrahepatic IVC and single lumen return cannula in the main pulmonary artery, or as a single, dual-lumen cannula (e.g., ProtekDuo^®^) (Figure 1). In patients with acute cor pulmonale, V-P ECMO has been shown to improve RV function, decrease vasoactive inotropic score, and improve oxygenation in patients with ARDS, likely by eliminating recirculation and uncoupling the dependence of oxygen delivery (DO_2_) on RV function [12,13,14,15]. V-P ECMO as an optimal configuration strategy, though, deserves further research to evaluate patient outcomes.

## 3. Physiology of Extracorporeal Membrane Oxygenation

The ECMO circuit comprises one or more cannulas (often one for drainage and one for return), a blood pump, a membrane lung, connectors, tubing, circuit monitoring, and temperature control (Figure 1). This system is designed to fully support the function of the failing respiratory system. Deoxygenated blood enters the extracorporeal circuit by removal from the intrahepatic inferior vena cava (IVC) via a drainage cannula (typical cannula size ranges 25 to 29 French) and a blood pump. Blood is then pumped through the membrane lung, where it is saturated with oxygen and carbon dioxide (CO_2_) is removed. The oxygen supply from the membrane lung is dependent on flow through the membrane lung, hemoglobin concentration, and the difference in oxygen content between the inlet and outlet. Because outlet blood is typically 100% saturated and the partial post-membrane lung pressure of oxygen (P_post-ML_O_2_) is typically > 500 mmHg, dissolved oxygen can be as much as 10% of oxygen content [16]. Oxygenated blood is then returned to the right side of the heart via a return cannula [16]. The final DO_2_ will be the mixture of native and ECMO flows with their respective oxygen contents from the native and membrane lungs multiplied by the total flow, which is a summation of native and extracorporeal venous flows. In V-V ECMO, mixing occurs in the right atrium. This can be evaluated using the following equation below (Equation), where C_3_ is the total oxygen content of the mixture, C_1_ is the oxygen content of circulation through the native lung, Flow_1_ is the native venous flow, C_2_ is the post-oxygenator oxygen content of the ECMO circuit, Flow_2_ is the ECMO venous flow, and Flow_Total_ is the summation of native and ECMO venous flows (or cardiac output): $Equation:C_{3}=\frac{C_{1}\times {Flow}_{1}}{{Flow}_{Total}}+\frac{C_{2}\times {Flow}_{2}}{{Flow}_{Total}}$.

DO_2_ for a patient on V-V ECMO will subsequently be dependent on hemoglobin concentration, oxygen saturation of hemoglobin, cardiac output, and the ratio of ECMO flow to cardiac output (Q_ECMO_/Q_CO_). This ratio can decrease, for example, in patients with high output states such as sepsis, and result in a decrease in DO_2_. An important concept in ECMO physiology is the ratio of oxygen delivery to oxygen consumption (VO_2_), or DO_2_:VO_2_. Average VO_2_ in healthy individuals may range between 3–4 mL/kg/min. However, in critically ill patients, VO_2_ may be increased under metabolic demands such as fever, pain, respiratory distress, inflammation, and increased catecholamines [17]. Conversely, VO_2_ may also be decreased in the setting of sepsis, significant multi-organ failure, and mitochondrial dysfunction [18,19]. Therefore, only by direct measurement can the VO_2_ be accurately assessed in critically ill patients. Under normal physiologic conditions, the DO_2_:VO_2_ ratio in adults is about 5:1. Anaerobic metabolism tends to occur when DO_2_:VO_2_ falls below 2:1. Ideally, when a patient is supported with ECMO for respiratory failure, the goal DO_2_:VO_2_ should remain ≥3:1 to provide some buffer to maintain aerobic metabolism. Though estimated DO_2_:VO_2_ can be calculated at the bedside, serum lactate may also be used as a surrogate to detect anaerobic metabolism. Typically, a Q_ECMO_/Q_CO_ of 0.6 will maintain an arterial oxygen saturation (S_a_O_2_) > 90% [20]. S_a_O_2_ > 80–85% is often sufficient to meet metabolic needs, though lower saturations may also be acceptable if DO_2_:VO_2_ remains adequate and there are no signs of tissue hypoxia [16,21].

## 4. Patient Selection

While ECMO remains a pivotal tool in managing select cases of acute respiratory failure, many of which are due to ARDS, the criteria for patient selection and optimal timing of implementation remain a focus of ongoing debate. Prior studies indicate varying outcomes when comparing ECMO to conventional ARDS management [4,5,22]. The most noteworthy prospective, randomized trials to date are the CESAR trial and the EOLIA trial [23,24]. The CESAR trial included patients with a Murray score ≥ 3 or a pH < 7.2 despite optimal ventilator settings. Patients were randomly assigned to conventional management or transfer to an ECMO center, though 20% of patients transferred to an ECMO center did not actually receive ECMO after optimization of mechanical ventilation. Of these patients, 82% survived. The CESAR trial demonstrated an overall survival benefit (63% versus 47%) for patients transferred to an ECMO center [23].

The EOLIA trial enrolled subjects meeting the following criteria: The ratio of partial pressure of arterial oxygen (P_a_O_2_) to the fraction of inspired oxygen (FIO_2_) (P/F) < 50 for more than 3 h or <80 for more than 6 h (with FIO_2_ > 80%) despite optimal ventilator settings and adjunctive measures such as paralysis, proning, and inhaled pulmonary vasodilators, or a pH < 7.25 and partial pressure of arterial carbon dioxide (P_a_CO_2_) > 60 mmHg despite optimal ventilator settings. Patients were randomized to receive ECMO or conventional management. Although the ECMO arm showed a trend toward reduced mortality, the trial utilized an intention-to-treat design. Notably, 28% of patients in the control group crossed over to receive salvage ECMO therapy, resulting in a 43% survival rate [24]. Post-hoc Bayesian analysis and meta-analysis suggested a potential ~10% mortality reduction with ECMO [25,26,27]. As a result, many ECMO centers use the EOLIA enrollment criteria when evaluating patient selection for ARDS. Other indications for ECMO may require more individualized criteria for patient selection. For example, life-threatening asthma is another common indication for ECMO support though there are not agreed upon criteria for cannulation. ECMO for life-threatening asthma carries relatively high survival rates. An ELSO registry study published in 2017 of patients with life-threatening asthma receiving ECMO demonstrated weaning success and hospital survival of 86.7% and 83.5%, respectively [28].

Absolute contraindications to ECMO include anticipated nonrecovery without a viable plan for ECMO decannulation (such as bridge to lung transplant), moribund patients with established multiorgan failure, poor short-term survival (such as metastatic malignancy), and catastrophic neurologic injury [4]. Relative contraindications include severe central nervous system injury or hemorrhage, irreversible and incapacitating central nervous system pathology, systemic bleeding, contraindications to anticoagulation, immunosuppression, older age (though no established threshold), and mechanical ventilation for more than 7 days [21]. While mechanical ventilation for more than 7 days is considered a relative contraindication to ECMO, and was an exclusion criterion in both CESAR and EOLIA trials, prolonged use of noninvasive respiratory support modalities in patients requiring high FIO_2_ and/or NIV may also be considered in deciding whether a patient is an appropriate candidate for ECMO. During the COVID-19 pandemic, prolonged use of high flow nasal oxygen (HFNO) and NIV prior to IMV were independently associated with mortality in patients that went on to require ECMO [29,30,31,32]. Obesity was previously thought to be a relative contraindication to ECMO, however, more recent data suggests that obese patients supported with ECMO have lower mortality risk and shorter ICU length of stay despite more device-related complications [33,34].

In addition to the above indications and contraindications, validated scoring models to predict mortality based on pre-ECMO factors exist to aid the clinician in patient selection. Validated models include the PRESERVE score [35], the PRESET score [36], and the RESP score (Table 2) [37]. The RESP score is commonly used and may benefit clinicians as a decision-making tool, though was validated prior to the COVID-19 pandemic. The COVID-19 pandemic produced mixed results in predicting mortality in patients with COVID-19-associated ARDS [38,39]. This may in part be due to the increased use of NIV and high flow nasal oxygen prior to IMV and the subsequent effects on mortality in patients that end up receiving ECMO support [29,30,31]. Factoring days spent on NIV and high flow nasal oxygen with days spent on IMV may provide improved prediction of the RESP score in patients with COVID-19-associated ARDS [32].

## 5. Ventilator Management and Adjunctive Therapies

**Figure 2:**
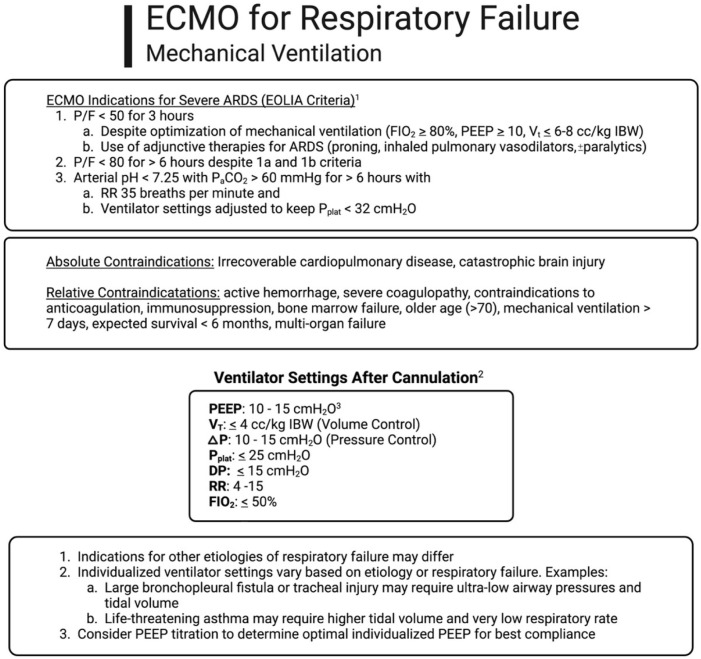
Algorithm demonstrating criteria for ECMO consideration, mechanical ventilator settings, and contraindications to ECMO for respiratory support. Abbreviations: acute respiratory distress syndrome, ARDS; driving pressure, DP; extracorporeal membrane oxygenation, ECMO; fraction of inspired oxygen, FIO_2_; ideal body weight, IBW; inspiratory pressure above PEEP, ∆P, partial pressure of arterial carbon dioxide (mmHg), P_a_CO_2_; positive end expiratory pressure, PEEP; ratio of partial pressure of arterial oxygen (mmHg) to FIO_2_, P/F; plateau pressure, P_plat_; tidal volume, V_T_; respiratory rate, RR. Adapted from Grotberg et al. [50].

Optimal ventilator settings for patients receiving ECMO for respiratory failure is an ongoing topic of debate. In general, V-V ECMO allows for “lung rest” with significant reductions in tidal volumes, driving pressure, plateau pressure (P_plat_) and mechanical power, which may reduce ventilator-induced lung injury (VILI) [40,41,42,43,44] (Figure 2). Much of the research in optimal ventilator settings is in the context of underlying ARDS. Higher positive end-expiratory pressure (PEEP) and lower driving pressure while on ECMO for ARDS has been associated with improved survival, and the use of electrical impedance tomography to set optimal PEEP suggests most patients require a PEEP between 10 and 15 cmH_2_O to minimize overdistension and atelectasis and improve pulmonary compliance [40,45,46,47,48,49]. Other etiologies of respiratory failure requiring ECMO may require more individualized ventilator settings. For example, a patient with tracheal injury or severe air leak syndrome from bronchopleural fistula may benefit from lower PEEP and ultra-lung rest settings (V_T_ ≤ 4 mL/kg IBW) to minimize pressure and flow in the native lung to promote wound healing. Patients with life-threatening asthma who require ECMO support (predominantly for extracorporeal CO_2_ removal) may benefit from higher tidal volumes, if plateau pressure (P_plat_) is maintained <30 mmHg, and ultra-low respiratory rates to facilitate adequate emptying of the lungs to avoid progressive auto-PEEP.

While adjunctive therapies, such as neuromuscular blockade, inhaled pulmonary vasodilators and prone positioning, are commonly used in mechanically ventilated patients with severe ARDS prior to ECMO cannulation, current evidence does not support the routine use of adjunctive therapies in patients receiving ECMO for ARDS. Of these mentioned, only prone positioning has ever demonstrated a significant reduction in mortality [51]. However, while observational evidence has suggested a benefit to prone positioning for patients receiving ECMO for ARDS [52], a recent randomized trial of 170 patients did not demonstrate a significant difference in mortality or time to ECMO weaning [53]. The routine use of inhaled pulmonary vasodilators and neuromuscular blockade has not been adequately studied in patients receiving ECMO for ARDS. The use of continuous neuromuscular blockade has not demonstrated any association with improved outcomes [54]. The use of these adjunctive therapies may be considered on an individual basis. Patients with high native pulmonary blood flow despite efforts to increase circuit flow can develop refractory hypoxemia and may benefit from adjunctive therapies to improve pulmonary ventilation-perfusion matching and oxygenation.

## 6. Management and Troubleshooting of the Extracorporeal Circuit

### 6.1. Physiologic Goals and Monitoring

Daily monitoring of the patient and extracorporeal circuit is vital to management of ECMO. Briefly, venous drainage cannulas should be examined for securement, signs of air, cracks, bleeding, fibrin/clot, tube chatter and color (dark red). The membrane lung should be examined for signs of air, clot/fibrin, and condensation. Condensation can be removed by “sigh-ing” the membrane lung. This is accomplished by increasing the sweep gas flow to 9–10 L/min for about 30 s. The return cannula should be examined for securement, signs of air, cracks, bleeding, fibrin/clot, tube chatter and color (bright red). The ECMO console should be routinely assessed for pump speed, ECMO blood flow, internal pressure and pressure gradient across the membrane lung (∆P), and pressures in the drainage and return cannulas. Normal values for ∆P are ~25 mmHg. The fraction of delivered oxygen (F_d_O_2_) and sweep gas flow should be monitored and adjusted as appropriate. Patient vital signs, volume status, and urine color (as a marker of hemolysis) should also be evaluated routinely. A hematologic profile should also be routinely monitored for signs of hemolysis and coagulopathy, including hemoglobin (Hb) and hematocrit (Hct), platelet count, fibrinogen, activated partial thromboplastin time (aPTT), prothrombin time (PT)/international normalized ratio (INR), lactate dehydrogenase (LDH) and plasma free hemoglobin (pfHb). Laboratory signs of hemolysis may include LDH > 2000 units/L or pfHb > 50 mg/dL. Elevated carboxyhemoglobin levels may be another indicator of hemolysis and are associated with higher mortality [55].

In general, oxygenation goals while on ECMO respiratory support differ from IMV. Typically, S_a_O_2_ > 80–85% is often acceptable in the setting of adequate DO_2_ and Q_ECMO_/Q_CO_. While a patient requiring IMV with a saturation of 80% is on the steep portion of the oxyhemoglobin dissociation curve and at risk of rapidly desaturating to more dangerous levels, a patient on ECMO has hemoglobin directly saturated by the sweep gas from the membrane lung and has larger dissolved oxygen buffer. Patients should be monitored for signs of tissue hypoxia (i.e., serum lactate, urine output, cyanosis or mottling of the skin). Sweep gas flow should target a P_a_CO_2_ to maintain pH ≥ 7.3, though in select patients, more permissive hypercapnia may be acceptable or desired. In patients with severe hypercapnia present prior to ECMO cannulation, P_a_CO_2_ should be gradually reduced using stepwise increments of sweep gas flow over 24 h. A relative reduction in P_a_CO_2_ > 50% ((Pre-ECMO P_a_CO_2_–Post-ECMO P_a_CO_2_)/Pre-ECMO P_a_CO_2_) increases the risk of neurologic complications [56,57]. Historically, Hb targets with ECMO were about 10 g/dL, however, evidence suggests that a Hb threshold of 7 g/dL is adequate for most patients [58,59].

Routine chest X-rays should be monitored for changes in underlying parenchymal disease, endotracheal or tracheostomy tube position, ECMO cannula position, and new pulmonary pathologies (e.g., pneumothorax, pleural effusions). The mechanical ventilator should be evaluated for respiratory rate (RR), V_T_, P_plat_, PEEP, driving pressure, and static lung compliance (C_stat_). Patients with worsening C_stat_ may have progression of their underlying disease, nosocomial infection, pneumothorax, or may need bronchial hygiene with therapeutic fiberoptic bronchoscopy, which has been shown to be safe and well tolerated in patients receiving ECMO [60]. Patients with improving C_stat_ may be showing signs of readiness for ECMO weaning.

Given the high prevalence of acute cor pulmonale in patients with severe ARDS, patients should be routinely monitored (e.g., weekly) with point-of-care echocardiography to monitor RV and LV function. Patients who develop RV dysfunction may require inotropes, inhaled pulmonary vasodilators, or reconfiguration to V-VA or V-P ECMO, while those who develop LV dysfunction may require inotropes or reconfiguration to V-A or V-VA ECMO.

### 6.2. Circuit Pharmacology

The ECMO circuit, which consists of conduit tubing and the membrane lung, creates a large surface area on which drugs can be adsorbed, particularly lipophilic drugs with high protein binding, resulting in an increase in the volume of distribution for many drugs, and thus decrease in serum drug concentrations. However, over time the adsorption phenomenon may decrease due to saturation of binding sites, and serum drug concentrations may increase over time resulting in toxicity. In some cases, the circuit may serve as a reservoir and redistribute drug even after it has been discontinued [61]. For example, sedatives with high circuit sequestration include fentanyl, midazolam, dexmedetomidine, and propofol, while those with lower circuit sequestration include ketamine, hydromorphone, morphine, oxycodone, and quetiapine [61,62,63,64]. These factors should be taken into account when choosing a sedation plan for patients receiving ECMO. For example, hydromorphone-based sedation in ECMO has been shown to result in more days alive without delirium or coma and in decreased narcotic requirements when compared to fentanyl-based sedation [65]. The ECMO circuit may also affect serum drug levels of antimicrobials. While the discussion of individual antimicrobials is beyond the scope of this review, most antimicrobials may be dosed at usual dosing strategies for critically ill patients based on weight and renal function as indicated. Other agents that have high sequestration, such as voriconazole, may require increased loading and maintenance doses [61]. When deciding an antimicrobial treatment plan, clinicians should weigh individual patient factors such as ECMO circuit factors, organism, site of infection, and antimicrobial choice.

### 6.3. Hypoxemia and Tissue Hypoxia

**Figure 3:**
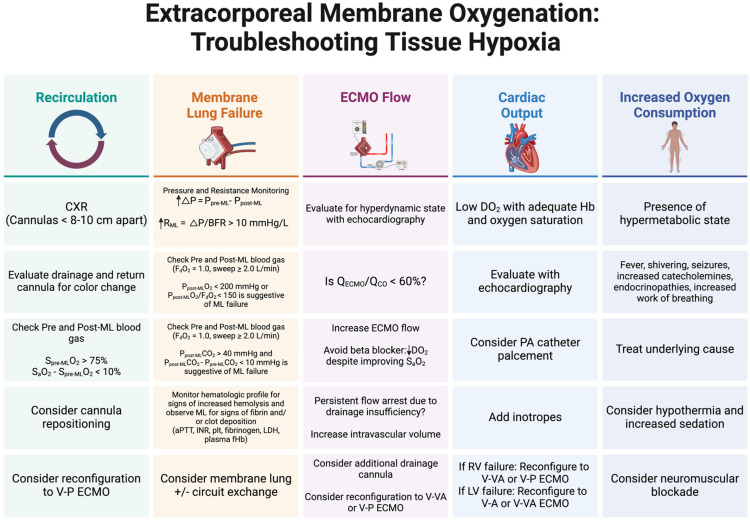
Algorithm for the diagnosis and management of common causes of hypoxemia and tissue hypoxia when receiving ECMO for respiratory failure. Abbreviations: activated partial thromboplastin time, aPTT; blood flow rate, BFR; chest X-ray, CXR; oxygen delivery, DO_2_; fraction of oxygen delivered to the sweep gas flow, F_d_O_2_; international normalized ratio, INR; lactate dehydrogenase, LDH; membrane lung, ML; pulmonary artery, PA; pre-membrane lung pressure, P_pre-ML_; post-membrane lung pressure, P_post-ML_; difference in pre- and post-membrane lung pressures, ∆P; partial pressure of oxygen in the venous drainage cannula, P_pre-ML_O_2_; partial pressure of oxygen in the return cannula, P_post-ML_O_2_; partial pressure of carbon dioxide in the venous drainage cannula, P_pre-ML_CO_2_; partial pressure of carbon dioxide in the return cannula, P_post-ML_CO_2_; platelets, plt; plastma free hemoglobin, pfHb; ECMO venous blood flow, Q_ECMO_; systemic cardiac output Q_CO_; oxygen saturation of arterial blood, S_a_O_2_; oxygen saturation of blood in the venous drainage cannula, S_pre-ML_O_2_; veno-pulmonary, V-P; veno-venoarterial, V-VA; oxygen consumption, VO_2_.

Hypoxemia and/or tissue hypoxia while on ECMO may occur under many conditions and will often manifest as an increase in serum lactate and an S_a_O_2_ < 80–85% despite a F_d_O_2_ of 1.0. Common causes of hypoxemia may include recirculation, failure of the membrane lung, insufficient ECMO flows or low Q_ECMO_/Q_CO_, decreased cardiac output resulting in low DO_2_, or increased utilization of oxygen in hypermetabolic states (Figure 3). In the setting of persistent hypoxemia and/or tissue hypoxia while supported with ECMO for respiratory failure, the clinician should investigate the underlying cause to determine the appropriate solution. The patient should be examined for signs of cyanosis, poor perfusion, or hypermetabolic state (e.g., shivering, seizures). The circuit should be examined for color change between the drainage and return cannulas and for signs of chatter or suck down resulting in little to no ECMO flow. The membrane lung should be examined for signs of fibrin deposition or thrombosis. A chest X-ray, pre- and post-membrane lung blood gases, and point-of-care echocardiogram should be obtained. Figure 3 shows methods by which causes of hypoxemia and/or tissue hypoxia may be diagnosed and treated. Increasing pressure gradient across the membrane lung with declining post-membrane lung partial pressure of oxygen (P_post-ML_O_2_) and/or rising P_post-ML_CO_2_ may be indicative of a failing membrane lung that requires exchange. If Q_ECMO_/Q_CO_ declines, usually in the setting of hyperdynamic left ventricular function, ECMO flow can be increased as long it does not exceed the rated flow of the membrane lung. While treatment with beta-blockers may improve Q_ECMO_/Q_CO_ and improve S_a_O_2_, they may also decrease DO_2_ and paradoxically worsen tissue hypoxia, and thus should generally be avoided [66]. ECMO flow may also decline in the setting of intravascular hypovolemia, intra-abdominal hypertension, or increased thoracic pressures (e.g., pneumothorax) and ECMO flow may arrest due to suck-down events. Intravascular volume expansion may improve ECMO flow and decrease flow arrest. Conversely, if the oxygenation allows for it, reducing ECMO flow may also alleviate further repetitive flow arrests (suck-down events). In select cases, adding an additional drainage cannula or reconfiguration to V-P ECMO may be required [15]. Alterations in the DO_2_:VO_2_ may also result in tissue hypoxia. In cases of low DO_2_ secondary to low cardiac output, point-of care echocardiography is useful in determining left ventricular (LV) and right ventricular (RV) performance which may respond to the addition of inotropic support. In select cases, reconfiguration to V-VA ECMO, or specifically in the case of RV failure, V-P ECMO may be required. Hypermetabolic states resulting in increased VO_2_ may be addressed on an individualized basis and may require increased sedation, or in severe cases, neuromuscular blockade (Figure 3).

Recirculation is a phenomenon that occurs when post-membrane oxygenated blood from the return cannula does not enter the right atrium, but rather enters the venous drainage cannula resulting in an increased oxygen saturation of pre-membrane lung blood (S_pre-ML_O_2_). This results in a higher proportion of de-oxygenated blood entering the right atrium and subsequently right ventricle, pulmonary circulation and systemic circulation leading to a decreased S_a_O_2_ [67]. This may occur in a bi-caval dual cannula V-V ECMO strategy when the drainage and return cannulas are positioned too closely together. Increasing ECMO flows can exacerbate recirculation, as increased negative pressure in the venous drainage cannula will pull more oxygenated blood from the return cannula. Cannula position should be evaluated by chest X-ray and drainage and return cannulas should be examined for color change, as cannulas < 8–10 cm apart with lack of color change may indicate recirculation. A S_pre-ML_O_2_ > 75% and/or S_a_O_2_–S_pre-ML_O_2_ < 10% also suggest recirculation. Recirculation may be ameliorated by repositioning drainage and return cannulas farther apart, or reconfiguring to a single, dual lumen cannula or V-P ECMO (Figure 3) [68].

### 6.4. Complications

Complications with ECMO are common, and in some cases, may be life-threatening. Cannulation complications occur in ~6% of ECMO cases for respiratory failure and may include vascular injury, retroperitoneal or cardiac injury, air embolism, infection, and venous thrombosis [4]. Cannula problems, air emboli, and thrombosis occur at rates of approximately 0.155, 0.025 and 0.103 per 1000 ECMO hours, respectively, in adults receiving ECMO for respiratory support [8]. Cannula site bleeding occurs at rates of approximately 0.123 per 1000 ECMO hours [8]. Cannulation should be performed by experienced operators with ultrasound or fluoroscopic guidance with adequate blood products available. Technical failure of the circuit or membrane lung (as described above) may also occur. Approximately 30% of patients may require ECMO system exchange (circuit and/or membrane lung) [69]. Recently, it has been noted that extracorporeal circuit exchange occurs at a rate of 0.258 per 1000 ECMO hours [8]. Other complications include but are not limited to bleeding from non-cannula sites such as the GI tract, central nervous system hemorrhage or ischemia, hemolysis, limb ischemia, and renal replacement therapy [4,8].

## 7. Weaning of Extracorporeal Support

**Figure 4:**
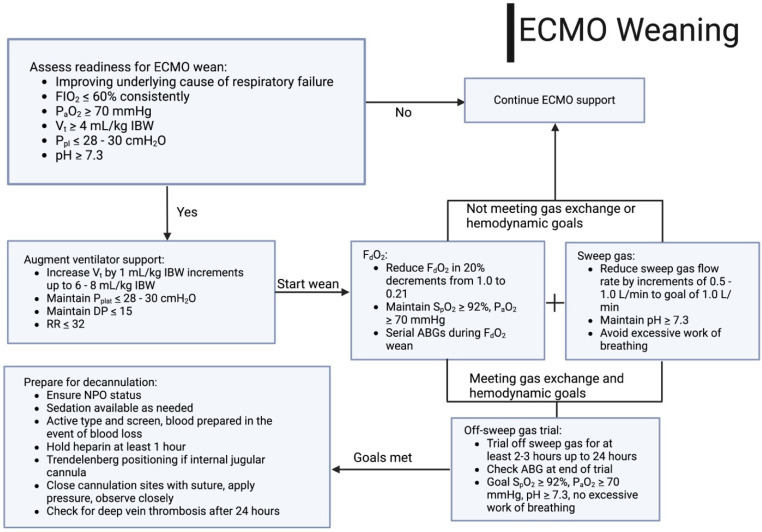
Algorithm demonstrating weaning strategies for ECMO for respiratory failure. Abbreviations: arterial blood gas, ABG; driving pressure, DP; extracorporeal membrane oxygenation, ECMO; fraction of oxygen delivered to the sweep gas flow, F_d_O_2_; fraction of inspired oxygen, FIO_2_; ideal body weight, IBW; arterial partial pressure of oxygen (mmHg), P_a_O_2_; arterial pressure of carbon dioxide (mmHg), PaCO_2_; positive end expiratory pressure, PEEP; plateau pressure, P_plat_; oxygen saturation by pulse oximetry, S_p_O_2_; tidal volume, V_T_; respiratory rate, RR.

Similar to weaning from IMV, the most important first step to weaning from ECMO support is resolution of the underlying insult to the lung. Signs of recovery may include improving pulmonary compliance, improving opacities on chest X-ray, increasing P_a_O_2_ and S_a_O_2_, and decreasing P_a_CO_2_ with increasing end-tidal CO_2_. Additionally, patients should be as close to euvolemic as possible, which may be accomplished with diuretics or volume removal by dialysis, to optimize the weaning process. V-V ECMO support can be reduced by decreasing the flow through the ECMO circuit (thereby increasing native venous flow through lungs), or by decreasing the oxygen content and sweep gas delivered to the membrane lung. Typically, if the patient is tolerating the current ECMO flows, these are held relatively constant and oxygen/sweep gas is weaned from the membrane lung [70]. F_d_O_2_ is gradually weaned in increments of 20% from 1.0 to 0.21 and the sweep gas is weaned by increments of 0.5–1.0 L/min to a goal of 1 L/min. However, it should be noted that not all centers wean ECMO F_d_O_2_, and instead will simply wean sweep gas by itself. Both strategies will ultimately accomplish the same goal. Ventilator settings are concurrently increased as well to support the ECMO wean, though still maintaining lung protective ventilation of 6–8 mL/kg IBW, plateau pressures less than 28–30 cmH_2_O, and FIO_2_ ≤ 60%. If an arterial blood gas remains acceptable (typically pH ≥ 7.3), then a “sweep off” trial is recommended, where the sweep is turned to 0. The “sweep off” trial may range from 2 to 24 h. The patient is monitored for signs of respiratory distress and hemodynamic instability, and arterial blood gases are monitored for worsening oxygenation (goal P_a_O_2_ ≥ 70) or respiratory acidosis (goal pH ≥ 7.3). If a patient tolerates this, then ECMO can be removed [21,71,72,73] (Figure 4). Just as in mechanical ventilation with spontaneous breathing trials, non-physician-driven weaning protocols for ECMO and “sweep off” trials have been shown to decrease ECMO days and hasten ECMO weaning and decannulation [74].

## 8. Summary

ECMO remains an integral mode of support for select patients with respiratory failure refractory to conventional IMV. Knowledge of patient selection, ECMO physiology, circuit management, and troubleshooting hypoxemia and other complications is vital for bedside clinicians. Lung rest settings should be used for patients with ARDS and individualized PEEP should be considered. Other pulmonary pathologies may require more individualized ventilator management. Patients should be monitored routinely for signs of right heart failure that may necessitate reconfiguration to V-P or V-VA ECMO to support the RV and hemodynamics. Signs of improving pulmonary physiology, imaging, and gas exchange parameters should prompt consideration for ECMO weaning if appropriate.

## Figures and Tables

**Table 1:** Indications for use of extracorporeal membrane oxygenation for respiratory failure.

**Acute Respiratory Distress Syndrome (ARDS)**
*Primary Pulmonary*
Infection
Aspiration Inhalational Injuries Blunt Pulmonary Trauma Ischemic–Reperfusion Injury Drug Toxicities
*Extrapulmonary* Sepsis Pancreatitis Severe Traumatic Shock
Transfusion-Related Acute Lung Injury (TRALI)
**Obstructive Lung Diseases**
Life-Threatening Asthma Chronic Obstructive Pulmonary Disease
Acute Eosinophilic Pneumonia
Acute Interstitial Pneumonia
Diffuse Alveolar Hemorrhage (DAH)
Acute Chest Syndrome
Thoracic Trauma
Parenchymal Lung Injury
Tracheal Injury
Bronchopleural Fistula
Peri-Lung Transplant
Bridge to Transplant
Primary Graft Dysfunction

**Table 2:** Calculation of RESP scoring.

RESP Score Parameter	Score
Age, yr	
*18–49*	0
*50–59*	−2
*≥60*	−3
Immunocompromised status	−2
Mechanical ventilation prior to the initiation of ECMO	
*<48 h*	3
*48 h to 7 d*	1
*>7 d*	0
Acute respiratory diagnosis group	
*Viral pneumonia*	3
*Bacterial pneumonia*	3
*Asthma*	11
*Trauma and burn*	3
*Aspiration pneumonitis*	5
*Other acute respiratory diagnosis*	1
*Non-respiratory and chronic respiratory diagnoses*	0
Central nervous system dysfunction	−7
Acute associated non-pulmonary infection	−3
Neuromuscular blockade agents before ECMO	1
Nitric oxide use before ECMO	−1
Bicarbonate infusion before ECMO	−2
Cardiac arrest before ECMO	−2
P_a_CO_2_, mmHg	
*<75*	0
*≥75*	−1
Peak inspiratory pressure, cmH_2_O	
*<42*	0
*≥42*	−1
Total Score	−22 to 15

Definition of Abbreviations: ECMO = extracorporeal membrane oxygenation.
